# Scratch-Based Isolation of Primary Cells (SCIP): A Novel Method to Obtain a Large Number of Human Dental Pulp Cells Through One-Step Cultivation

**DOI:** 10.3390/jcm13237058

**Published:** 2024-11-22

**Authors:** Yuki Kiyokawa, Masahiko Terajima, Masahiro Sato, Emi Inada, Yuria Hori, Ryo Bando, Yoko Iwase, Naoko Kubota, Tomoya Murakami, Hiroko Tsugane, Satoshi Watanabe, Takahiro Sonomura, Miho Terunuma, Takeyasu Maeda, Hirofumi Noguchi, Issei Saitoh

**Affiliations:** 1 Department of Pediatric Dentistry, Asahi University School of Dentistry, Gifu 501-0296, Japan; ykiyokawa@dent.asahi-u.ac.jp (Y.K.); ped-yuria@dent.asahi-u.ac.jp (Y.H.); shounishika-bando@dent.asahi-u.ac.jp (R.B.); tomoya.ip@gmail.com (T.M.); hiroko@dent.asahi-u.ac.jp (H.T.); 2 Department of Anatomy, Asahi University School of Dentistry, Gifu 501-0296, Japan; terajima5708@gmail.com (M.T.); sonom@dent.asahi-u.ac.jp (T.S.); 3 Department of Genome Medicine, National Center for Child Health and Development, Tokyo 157-8535, Japan; sato-masa@ncchd.go.jp; 4 Department of Pediatric Dentistry, Graduate School of Medical and Dental Sciences, Kagoshima University, Kagoshima 890-8544, Japan; inada@dent.kagoshima-u.ac.jp (E.I.); kubotarecta@gmail.com (N.K.); 5 Department of Dentistry for the Disability and Oral Health, Asahi University School of Dentistry, Gifu 501-0296, Japan; iwase@dent.asahi-u.ac.jp; 6 Kyoto Dental Service Center Central Clinic, Kyoto 604-8418, Japan; 7 Institute of Livestock and Grassland Science, Ibaraki 305-0901, Japan; kettle@affrc.go.jp; 8 Division of Oral Biochemistry, Faculty of Dentistry, Graduate School of Medical and Dental Sciences, Niigata University, Niigata 951-8514, Japan; mterunuma@dent.niigata-u.ac.jp; 9 Center for Advanced Oral Science, Graduate School of Medical and Dental Sciences, Niigata University, Niigata 951-8514, Japan; maedat@dent.niigata-u.ac.jp; 10 Department of Regenerative Medicine, Graduate School of Medicine, University of the Ryukyus, Okinawa 903-0215, Japan; noguchih@med.u-ryukyu.ac.jp

**Keywords:** dental pulp, cell cultivation, scratch, primary cells, stem cells, human exfoliated deciduous teeth, cell-based regenerative medicine, cell resource dental pulp, regenerative medicine, connective tissue

## Abstract

**Background:** Dental pulp (DP) is a connective tissue composed of various cell types, including fibroblasts, neurons, adipocytes, endothelial cells, and odontoblasts. It contains a rich supply of pluripotent stem cells, making it an important resource for cell-based regenerative medicine. However, current stem cell collection methods rely heavily on the enzymatic digestion of dissected DP tissue to isolate and propagate primary cells, which often results in low recovery rates and reduced cell survival, particularly from deciduous teeth. **Methods:** We developed a novel and efficient method to obtain a sufficient number of cells through a one-step cultivation process of isolated DP. After the brief digestion of DP with proteolytic enzymes, it was scratched onto a culture dish and cultured in a suitable medium. By day 2, the cells began to spread radially from DP, and by day 10, they reached a semi-confluent state. Cells harvested through trypsinization consistently yielded over 1 million cells, and after re-cultivation, the cells could be propagated for more than ten passages. **Results:** The proliferative and differentiation capacities of the cells after the 10th passage were comparable to those from the first passage. The cells expressed alkaline phosphatase as an undifferentiation marker. Similarly, they also maintained the constitutive expression of stem cell-specific markers and differentiation-related markers, even after the 10th passage. **Conclusions:** This method, termed “scratch-based isolation of primary cells from human dental pulps (SCIP)”, enables the efficient isolation of a large number of DP cells with minimal equipment and operator variability, while preserving cell integrity. Its simplicity, high success rate, and adaptability for patients with genetic diseases make it a valuable tool for regenerative medicine research and clinical applications.

## 1. Background

Human deciduous dental pulp cells (HDDPCs) include stem cells obtained from human exfoliated deciduous teeth (SHEDs). SHEDs demonstrate a high colony-forming capacity, rapid proliferation rate, and the ability to differentiate into various lineages and cell types, such as osteocytes, adipocytes, and vascular endothelial cells, under suitable conditions [1,2,3,4,5,6,7,8,9,10]. They are believed to originate from neural crest cells [8] and share characteristics with mesenchymal stem cells and bone marrow stromal stem cells [11]. SHEDs exhibit high pluripotency and are known to differentiate into ectodermal, mesodermal, and endodermal lineages [12,13,14]. Despite these properties, SHEDs carry a low risk of oncogenesis and are considered safe [15,16,17]. SHEDs have gained considerable attention due to their non-invasive, painless collection from children, along with their low immunogenicity and the lack of related ethical concerns [18,19,20]. As a result, SHEDs are regarded as a promising material for cell-based regenerative medicine [20]. For example, osteogenesis was observed when SHEDs were implanted into injured skull regions in mice [21,22], and extensive bone regeneration was reported when SHEDs and dental pulp (DP) stem cells were transplanted into the calcaneus. Research is also aiming to regenerate cartilage, leveraging SHEDs’ ability to differentiate into chondrocytes [23]. Furthermore, studies have explored cranial nerve regeneration by creating neurospheres from SHEDs and transplanting them into rats with Parkinson’s disease [5,24], while attempts have been made to regenerate trigeminal nerves through local SHED transplantation [25]. In dentistry, efforts have focused on regenerating tooth roots by creating cell sheets from cultured SHEDs and transplanting them subcutaneously into the jawbone [26]. As demonstrated above, SHEDs have been increasingly utilized in various regenerative studies.

Gronthos et al. first reported the successful isolation and cultivation of these cells approximately 20 years ago [27,28]. According to their research, dissected DP cells were initially digested with enzymes such as collagenases (at 37 °C for over an hour), followed by filtration through a cell strainer to purify single cells [1,27]. However, the number of recovered cells was limited (approximately 1 × 10^5^ cells after 2 weeks of cultivation), potentially due to damage caused by prolonged enzymatic treatment and strainer-mediated cell sorting [29]. In addition, DP cells isolated using the conventional method often lose their stem cell properties, as they tend to differentiate into osteoblasts and neurons with repeated passaging [29]. Telomere length reduction has also been observed in isolated SHEDs during long-term cultivation [30,31]. As HDDPCs are considered as crucial biomaterials for regenerative research [20], it is crucial to prepare a large quantity of non-damaged HDDPCs to support their clinical application in regenerative medicine. However, there have been few studies aimed at improving efficient and stable methods for primary HDDPC culture.

In this study, we found that DPs could easily adhere to the surface of a plastic tissue culture dish by scratching the tissue with a scalpel, allowing for the repeated recovery of primary cells. We named this method the “scratch-based isolation of primary cells from human dental pulps” (SCIP). This study aimed to evaluate the applicability of the SCIP method in regenerative research. Investigations were conducted as follows: (1) the number of cells that can be recovered through a single trypsinization of semi-confluent cells, (2) the number of times this process can be repeated to collect a sufficient quantity of cells from the same sample, (3) whether cells can retain their pluripotency and proliferation efficiency after the repeated trypsinization of a DP, and (4) whether this approach is applicable to DPs from patients with dental diseases (such as prolonged retention, pulpitis, or occlusal trauma) and systemic diseases were determined.

## 2. Materials and Methods

### 2.1. Ethics Statement

All experiments were conducted in accordance with the relevant guidelines and regulations. The Ethics Committee of Asahi University approved the study protocol (approval number: 34007). Written informed consent was obtained from participants or their parents prior to participation in this study.

### 2.2. Extraction of DPs and Their Scraping onto the Culture Dish

The DPs were obtained from the deciduous teeth of patients with or without systemic diseases. The teeth were soaked in Minimum Essential Medium α (MEM α) (#135-15175; Wako Pure Chemical Industries, Osaka, Japan) containing 100 U/mL of penicillin–streptomycin (P/S) (#15140122; Thermo Fisher Scientific, Waltham, MA, USA) and 10% fetal bovine serum (FBS) (#10437028; Thermo Fisher Scientific), hereinafter referred to as MEMα/10% FBS. DPs were extracted using sterilized dental cleansers and tweezers. The time from pulp collection to primary culture was approximately 2–3 h. The extracted pulps were cut into approximately 1 mm pieces using a sterile scalpel (#37445000-No.11; FEATHER, Osaka, Japan) and placed onto a 35 mm gelatin-coated culture dish (#4000-020; Iwaki Glass Co., Ltd., Tokyo, Japan) containing 3 mg/mL collagenase type I (#17100017; Invitrogen, Carlsbad, CA, USA), 4 mg/mL dispase (#410810077; Roche Applied Science, Basel, Switzerland), and 1 mg/mL primocin (#ant-pm-05; InvivoGen, San Diego, CA, USA) in phosphate-buffered saline (PBS) (#1001049; Thermo Fisher Scientific). The dish was incubated for 15 min in an atmosphere containing 5% CO_2_ at 37 °C. The enzyme-treated DPs were then transferred to a new 35 mm gelatin-coated dish with 2 mL of MEMα/10% FBS containing 1 mg/mL primocin and scraped several times with a sterilized scalpel to promote sample attachment to the dish surface.

**Figure 1:**
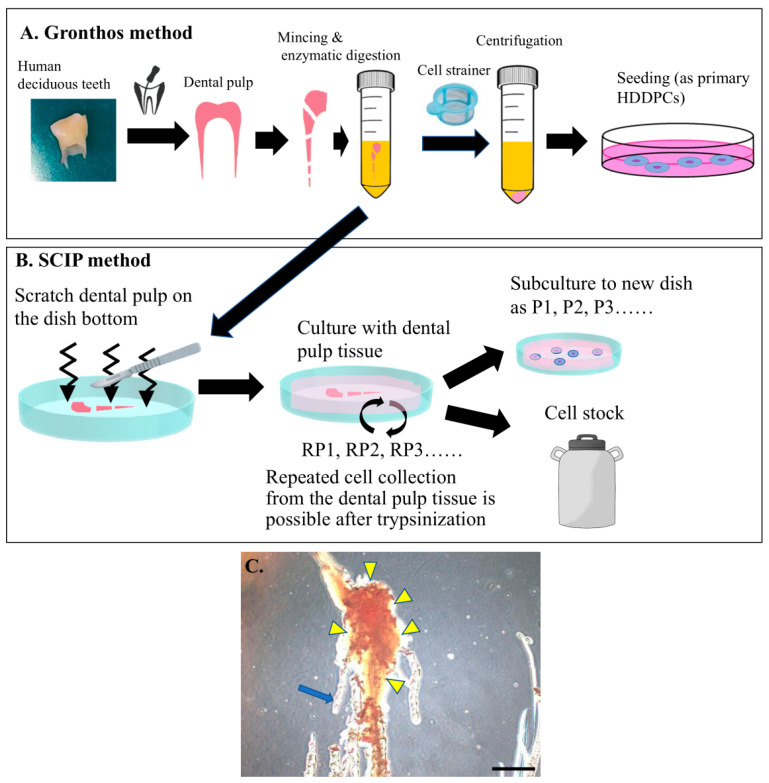
The conventional method and a new method (scratch-based isolation of primary cells from human dental pulps [SCIP]) were used for the isolation of dental pulp (DP) cells. (**A**) The conventional method was described by Gronthos et al. [27,28]. DP tissues were harvested from teeth, minced, and subjected to enzymatic digestion. The cells were then filtered through a cell strainer, centrifuged, and collected. (**B**) In the SCIP method used in this study, harvested DP tissues were minced and enzymatically digested for a short period. They were then scratched onto the surface of a plastic dish containing the growth medium. This method enabled us to obtain cells repeatedly, owing to the tight attachment of DP tissues to the bottom of the dish. After obtaining cells by trypsinization, the dish was replenished with fresh medium. This allowed cells to exhibit outgrowth from the DP tissues, making it possible to harvest them again. We call this repeated cell collection “repeated passage (RP)”. (**C**) The DP tissue was scraped from the bottom of the culture dish. The scar made by the scalpel on the dish surface is shown by an arrow. Arrowheads indicate DP tissue. Scale bar, 50 μm.

The strong attachment of the DPs to the dish was confirmed under a light microscope, ensuring that the cells did not float (Figure 1). The dish was incubated in an atmosphere containing 5% CO_2_ at 37 °C, with the medium replaced twice weekly. After 9 days of cultivation, DPs were incubated in primocin-free MEMα/10% FBS. On day 10, semi-confluent cells were harvested via trypsinization by incubating them in a solution containing 0.25% trypsin (#15090046; Thermo Fisher Scientific) in PBS for 5 min at 37 °C. The collected cells were counted and cryopreserved using Cell Banker (#CB021; TaKaRa Bio Inc., Shiga, Japan). An additional 2 mL of MEMα/10% FBS was added to the DP-containing dish to promote further cell outgrowth. Trypsinization and cell storage were repeated at least ten times.

During cultivation, the cells within the DP are released and begin migrating away, a process known as “cell outgrowth”. This outgrowth continues unless the migrating cells experience contact inhibition due to cell-to-cell contact. When this inhibition is alleviated by trypsinization, active outgrowth resumes. Therefore, repeated trypsinization can theoretically yield a large number of cells from a single DP, as long as the DP continues to provide cells. This method, in which the collected pulp is continuously cultured by scraping it onto the dish surface, is termed “scratch-based isolation of primary cells from human dental pulps” (SCIP).

### 2.3. Alkaline Phosphatase (ALP) Assay

Cells at semi-confluency (80–90% confluence) in a 35 mm gelatin-coated culture dish were washed twice with PBS and then fixed by incubating them with 4% paraformaldehyde for 5 min at 24 °C. After two additional PBS washes, the fixed cells were stained using an Alkaline Phosphatase Staining Kit II (#00-0055; Riprocell, Yokohama, Japan) for 15 min at 24 °C. Once stained, the cells were soaked in PBS, and phase images were captured using a microscope (#IX73; Olympus, Tokyo, Japan). The ALP-stained activity was analyzed with ImageJ software (version 1.53e), as performed in a previous study [32]. The cell images were changed to 8-bit and the gradation value of the cells was measured. The ALP signal intensity was calculated from the mean of the gradation value.

### 2.4. Reverse Transcription Polymerase Chain Reaction (RT-PCR) Analysis

Cells were first cultured in a 100 mm gelatin-coated dish with MEMα/10% FBS to examine the marker gene expression. Semi-confluent cells were harvested by trypsinization, and total RNA was isolated using an RNA mini kit (#50204; QIAGEN, Limburg, The Netherlands). cDNA was synthesized using the First Strand cDNA Synthesis Kit (#18080-051; Invitrogen). RT-PCR was performed using a SimpliAmp Thermal Cycler (#A24811; Applied Biosystems, Foster City, CA, USA) and specific primer sets (Table 1; Thermo Fisher Scientific). The resulting RT-PCR products were subjected to gel electrophoresis on a 2% agarose gel (#16500-100; Invitrogen) stained with SYBR Safe DNA Gel Stain (#S33102; Invitrogen). DNA bands were photographed under UV light.

### 2.5. Immunocytochemical Staining

The cells obtained after repeated passaging were cultured in a 35 mm gelatin-coated dish using MEMα supplemented with 10% FBS. The cells passaged at RP2 plus P4 and RP9 plus P4 were used for immunocytochemical staining. Semi-confluent cells were washed with PBS, fixed in 4% PFA for 5 min at 24 °C, and blocked via incubation in 20% AquaBlock (#PB82; East Coast Biologics, North Berwick, ME, USA) for 30 min at 24 °C. Proteins were probed with an anti-OCT3/4 antibody (#MAB4401; Sigma-Aldrich, St. Louis, MO, USA; 1:200) or an anti-SSEA1 antibody (#ab16285; Abcam, Tokyo, Japan; 1:200). Alexa Fluor 594-conjugated goat anti-mouse IgM (#ab150116; Abcam; 1:200) was used as the secondary antibody, and nuclear staining was performed using 4′,6-diamidino-2-phenylindole (DAPI) (#H-1200; Vector Laboratories, Newark, CA, USA). Proteins were visualized using an Olympus IX73 fluorescence microscope (Olympus, Tokyo, Japan), and micrographs were captured with a digital camera (FUJIX HC-300/OL; Fujifilm, Tokyo, Japan).

### 2.6. Success Rates of the SCIP Method

Using the SCIP method, cells were isolated from the DP tissues obtained from patients with or without systemic and dental diseases. The cells underwent repeated passaging, and the success rates were evaluated based on the number of passages and cryopreserved cell stocks.

## 3. Results

### 3.1. Acquisition of HDDPCs by SCIP

**Figure 2:**
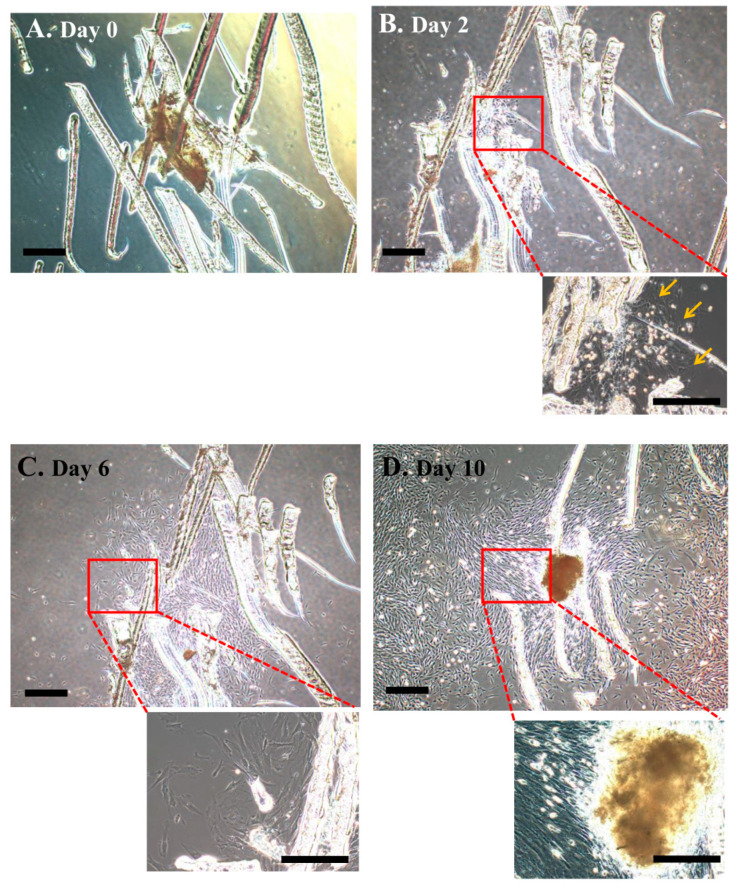
Cell proliferation in scraped DP tissues attached to the culture dish surface. (**A**) Image of the scraped DP tissues cultured in MEMα/10% FBS. Images were captured on the day of scratching (designated day 0). (**B**) Image of scraped DP tissue cultured on day 2. Cell outgrowth (arrows) was first observed. (**C**) Image of scraped DP tissues cultured on day 6. Cells proliferated radially around the DP tissue. (**D**) Image of scraped DP tissue cultured on day 10. The cells were allowed to reach confluence. Scale bar, 100 μm. DP cells from No. 12 were used (see Table 2 for details).

**Figure 3:**
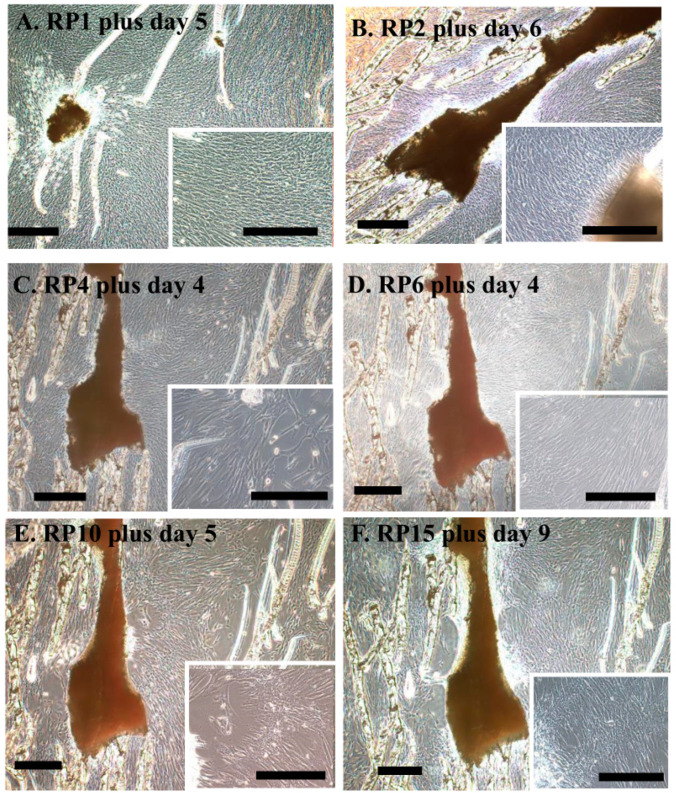
Time required to reach ≥ 80% confluence following repeated passage (RP). (**A**) Cells reached confluence on day 5 after RP1. (**B**) Cells reached confluence on day 6 after RP2. (**C**) Cells reached confluence on day 4 after RP4 treatment. (**D**) Cells reached confluence on day 4 after RP6 treatment. (**E**) Cells reached confluence on day 5 after RP10 treatment. (**F**) Cells reached confluence on day 9 of RP15 treatment. Scale bar, 100 μm. RP, repeated passage. DP cells from No. 12 were used (see Table 2 for details).

To test the usefulness of SCIP, the human DPs isolated from exfoliated deciduous teeth were subjected to enzymatic digestion, followed by scratching at the bottom of the culture dish, but no cells were observed initially (Figure 2A). By the second day of culture, the outgrowth of fibroblastic cells was discernible around the DPs attached to the plastic surface (Figure 2B). Subsequently, the cells grew radially from the DP tissue (Figure 2C). At this stage, no noticeable cell death was observed in the DP tissue. By the tenth day of culture, the cells surrounding the DP tissue had reached confluency (Figure 3D).

### 3.2. SCIP-Based Acquisition of HDDPCs Through Repeated Passage

Next, we examined whether cells could be efficiently collected following repeated passage (RP) (Figure 1B). Theoretically, as long as viable cells remain in the inner section of the DP, cells can continue to be obtained. We successfully collected a sufficient number of cells from DP tissue that had undergone 15 rounds of repeated passaging (RP15) (Figure 3F). The cells obtained at RP10 exhibited a fibroblast-like morphology, which was indistinguishable from that of cells obtained at RP1 (Figure 1A). However, the number of elongated cells increased in the population harvested at RP15 (Figure 3F).

**Figure 4:**
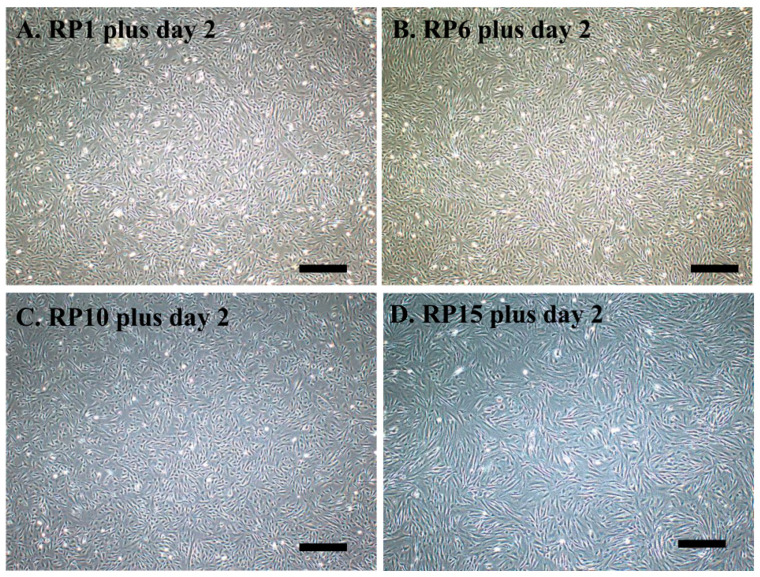
Subculture after repeated passage (RP). The cells obtained at (**A**) RP1, (**B**) RP6, (**C**) RP10, and (**D**) RP15 were re-plated onto 60 mm gelatin-coated culture dishes, and reached confluence 2 days after seeding. However, the number of cells derived from RP15 cells was low. Scale bar, 100 μm. DP cells from No. 12 were used (see Table 2 for details).

We also compared the time required to reach confluence between the cells obtained at RP1 and RP15 after trypsinization. There was little difference in the time needed for either group to reach 80% confluence, with both taking about 5 days. However, the cells obtained at RP15 tended to show a slight delay in reaching confluence (Figure 3F and Figure 4D).

### 3.3. Cytochemical Staining for ALP Activity

**Figure 5:**
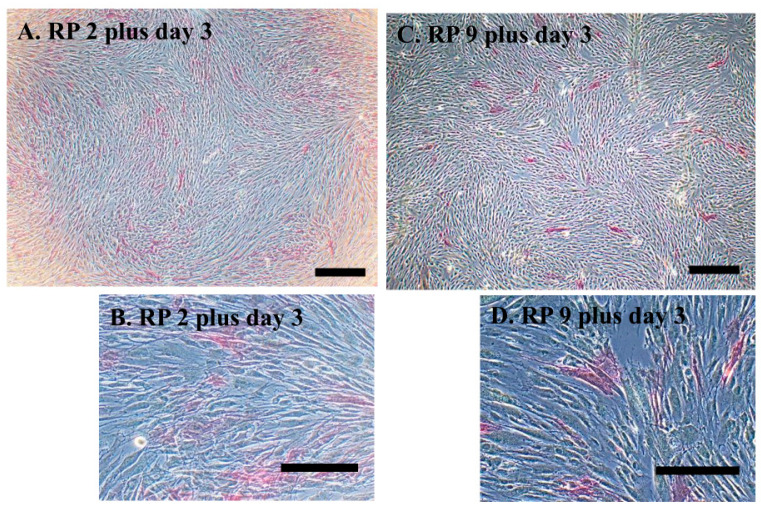
Cytochemical staining for alkaline phosphatase. (**A**,**C**) ALP-stained cells with phase images. Cells were subjected to ALP staining three days after RP2 and RP9. (**B**,**D**) High-magnification-rate phase images. Scale bar, 100 μm. DP cells from No. 12 were used (see Table 2 for details).

ALP is known to be a useful marker for pluripotent cells, such as ES cells and iPS cells [33,34], and we have previously demonstrated that a certain percentage of ALP-positive cells exist in HDDPCs [35]. The cells obtained at RP2 and RP9 exhibited ALP signals. The ALP signal strength was 72.7 bits for cells obtained at RP2 and 89.6 bits for those obtained at RP9. These results suggest that ALP activity was well maintained even in cells obtained at RP9 (Figure 5).

### 3.4. RT-PCR Analysis

**Figure 6:**
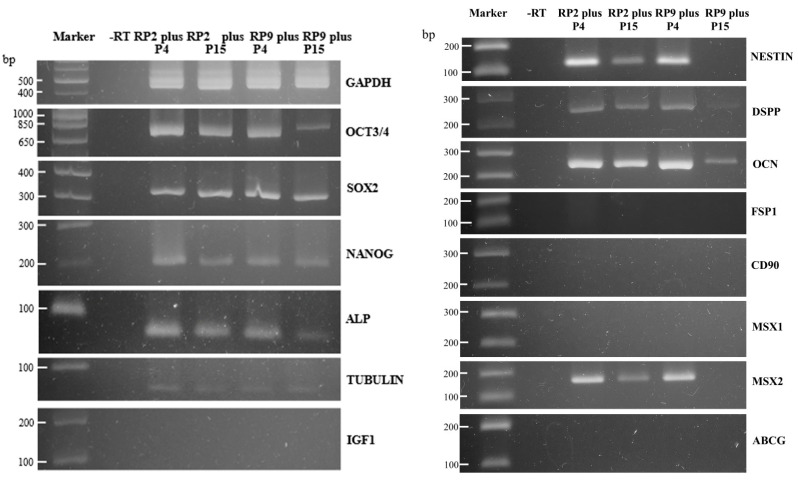
RT-PCR analysis of stem cell-related gene expression in HDDPCs. The RT-PCR products obtained from HDDPCs that underwent four cell passages after PR2 are designated RP2 plus P4. Similarly, cells that underwent 15 passages after PR2 and 4 passages after RP9 or 15 passages after RP9 were designated as RP2 plus P15, RP9 plus P4, and RP9 plus P15, respectively. GAPDH, glyceraldehyde-3-phosphate dehydrogenase; OCT3/4, octamer-binding protein 3/4; SOX2, SRY-box transcription factor 2; NANOG, Nanog homeobox; ALP, Alkaline phosphatase; TUBULIN, Tubulin; IGF1, insulin-like growth factor 1; NESIN, nestin; DSPP, dentin sialophosphoprotein; OCN, osteocalcin; FSP1, fibroblast-specific protein 1; CD90, cluster of differentiation 90; MSX1, Msh homeobox 1; MSX2, Msh homeobox 2; ABCG, ATP-binding cassette subfamily G; -RT, negative control (water was used for PCR). DP cells from No. 12 were used (see Table 2 for details).

We analyzed the gene expression of the stemness markers in cells collected from SCIP using RT-PCR (Figure 6).

In this study, cells were harvested after 4 or 15 passages from those initially collected at RP2, referred to as RP2 plus P4 or RP2 plus P15, respectively. Similarly, RP9 plus P4 cells were defined as cells harvested after four passages from those collected at RP9. The expression levels of stem cell markers, including octamer-binding transcription factor 4 (OCT3/4), SRY-box transcription factor 2 (SOX2), Nanog Homeobox (NANOG), and ALP, were similar across the three groups (RP2 plus P4, RP2 plus P15, and RP9 plus P4). However, in RP9 plus P15 cells, the mRNA levels of OCT3/4 and ALP were reduced, suggesting that the prolonged cultivation of HDDPCs leads to a decrease in the number of undifferentiated stem cells. The levels of neuroepithelial stem cell protein (NESTIN; a neurogenic differentiation marker), dentin sialophosphoprotein (DSPP; an odontogenic differentiation marker), osteocalcin (OCN; an osteogenic differentiation marker), and MSX2 (Msh homeobox 2; a transcriptional regulator in bone development) remained comparable to those observed in RP9 plus P4, but decreased significantly in RP9 plus P15 cells. These results suggest that the expression of markers specific to HDDPCs was preserved in cells at least up to RP9 plus P4. Interestingly, tubulin mRNA (a key regulator of cell morphology and division) was consistently expressed in cells from RP9 plus P4 to RP9 plus P15. However, markers such as ferroptosis suppressor protein 1 (FSP1; a fibroblast marker), insulin-like growth factor 1 (IGF1; a growth factor similar to insulin), ATP-binding cassette subfamily G member 1 (ABCG; an epithelial stem cell marker), CD90 (Thy-1 membrane glycoprotein precursor; a marker for various stem cells), and Msh homeobox 1 (MSX1; a transcriptional repressor in embryogenesis) were not detected in HDDPCs.

### 3.5. Immunocytochemical Analysis

SSEA-1 (stage-specific embryonic antigen-1) and OCT3/4 are well-known markers specifically expressed in pluripotent cells such as ES cells and iPSCs [36]. The expression of these molecules in human DP cells has been previously reported [35,37].

**Figure 7:**
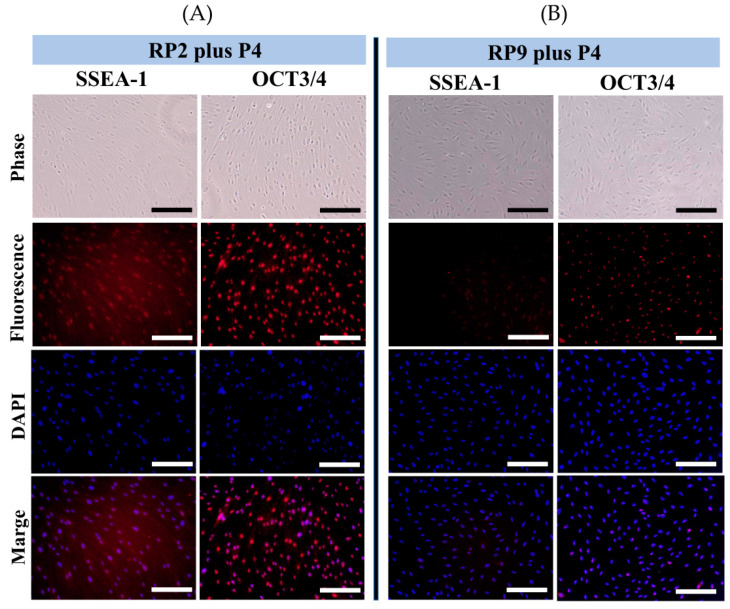
Immunocytochemical analysis of HDDPCs derived from RP2 plus P4 and RP9 plus P4 using anti-SSEA-1 (**A**) and anti-OCT3/4 antibodies (**B**). DAPI was used to visualize nuclear DNA. Bar, 100 μm. DP cells from No. 12 were used (see Table 2 for details).

When immunocytochemical analysis was performed on cells obtained after RP, the fluorescence intensity was higher in cells treated at RP2 plus P4 compared to those at RP9 plus P4 (Figure 7). In particular, a significant reduction in SSEA-1 expression was observed in cells treated at RP9 plus P4. However, OCT3/4 expression was maintained in both the RP2 plus P4 and RP9 plus P4 groups.

### 3.6. SCIP-Based Propagation of DP Cells from Patients

Next, we used the SCIP method to obtain cells from DP tissues derived from patients with various diseases. Of the 17 subjects, 14 had systemic diseases, including hypophosphatasia, osteogenesis imperfecta, Baller–Gerold syndrome, Rett syndrome, or Charge syndrome (Table 2). The dental findings included prolonged retention, tooth loss, or pulpitis. Among the 17 DP tissue samples tested, adequate amounts of primary DP cells were successfully isolated from 16 samples (Table 2), resulting in a success rate of over 94%.

For the pulp-1 to pulp-10 samples, more than five passages were possible, with approximately 30 cell stocks cryopreserved. For the pulp-11 to pulp-17 samples, the number of passages exceeded 15, and the maximum number of cryopreserved cell stocks was 50.

## 4. Discussion

The characteristics of primary cells can change with each subsequent passage if the initial isolation is inadequate or if the optimal culture conditions are not properly maintained. Several researchers have evaluated the proliferation rate of DPSCs isolated using the methods described by Gronthos et al. [7]. Suchánek et al. [38] showed that DPSCs derived from permanent DP tissues were successfully cultured, with the doubling time remaining unchanged even after the 18th passage, and no signs of degeneration or spontaneous differentiation were noted during cultivation. Similar observations were made with SHED cells by Suchánek et al. [9]. We found that the HDDPCs obtained by SCIP up to RP9 plus P15 exhibited no change in proliferation, suggesting the usefulness of SCIP for isolating and propagating HDDPCs. The limited cell yield from DPs in previous systems may be due to the fact that only a small number of cells can be isolated from the surface of a DP, which is easily accessible to enzymes. In contrast, the SCIP method requires less enzymatic treatment time, which may be advantageous for cell proliferation due to reduced damage to the cells.

The expression of marker genes specific to HDDPCs is also useful for characterizing primary cells. We found no alterations in ALP activity in cells obtained after RP9. Additionally, the mRNA levels of stem cell markers (OCT3/4, SOX2, and NANOG) remained unchanged until RP9 plus P4. These results suggest that DPs attached to the surface of a culture dish can provide stem cell-containing cells for an extended period (up to RP9 plus P4). Notably, the expression of stem cell markers, such as OCT3/4 and ALP, was well maintained in cells obtained at RP9 plus P4 but not at RP9 plus P15, suggesting that the number of cells with stem cell characteristics slightly decreases with continuous cultivation (repeated passages). Given that similar trends were observed in DP cells isolated using the method of Gronthos et al. [7], the decrease in stem cells with repeated passages may not be specific to the SCIP method itself. Furthermore, the expression of SSEA-1 was markedly decreased in cells at RP9 plus P4 compared to those isolated at RP2 plus P4 (Figure 7). However, OCT3/4 expression was maintained (Figure 6), suggesting that SSEA-1 may be a more sensitive marker for detecting primary cell naiveness.

DPSCs, such as stem cells in HDDPCs, have multiple potential applications. For example, under differentiation-inducing conditions, they can differentiate into various cell types, including neurons, osteoblasts, and adipose cells [35]. In our system, the mRNA levels of NESTIN (related to neurogenic events), DSPP (related to odontogenic events), and OCN (related to osteogenic events) remained unchanged up to RP9 plus P4, suggesting that the multilineage differentiation ability of DP cells isolated by SCIP was well preserved. NESTIN, in particular, is associated with the maintenance of hematopoietic stem cells [39], and its presence in SCIP suggests the maintenance of DP pluripotency. As cells passaged multiple times (i.e., P15) showed reduced cellular proliferation, the expression of these markers decreased with prolonged passages (Figure 2). This may be related to the decreased number of stem cells in the primary isolated DP cells.

When the conventional method described by Gronthos et al. [27,28] was used to isolate DP cells from both caries-bearing and healthy teeth, the success rates for acquiring primary cells were approximately 70% and 80%, respectively [40]. Similarly, Perry et al. reported a success rate of around 80% for primary pulp cell cultures [41]. Galler et al. suggested that the nonspecific inflammatory reactions of DP tissue against bacteria could reduce the number of stem cells, which may be related to the decreased success rates in primary cell culture [42]. In contrast, our SCIP-based method for isolating primary DP cells showed a high success rate (>94%; Table 2), even when the samples included DPs from teeth with pulpitis and occlusal trauma. Another advantage of the SCIP method is that no bacterial contamination was observed in the DP cell cultures, despite the known risk of yeast contamination during primary pulp cultures [41]. However, we were unable to isolate DP cells from patients with Baller–Gerold syndrome (Table 2). This failure could be attributed to a delay of over one day between tooth extraction and the initiation of SCIP. It is likely that the DP tissue was not preserved well prior to the initiation of primary cell culture, leading to reduced cell viability. Notably, previous studies have demonstrated that DP cell collection rates begin to decline as time elapses after pulp sampling [41].

Our SCIP method offers several advantages over conventional DP cell isolation methods (Table 3). For example, the SCIP method requires no special equipment beyond a scalpel and shows little operator variability. Cell damage is minimized by the short enzyme treatment period of only about 20 min. Cells grown as “outgrowth” can be easily harvested and cryopreserved. Additionally, this method can continuously provide DP cells and collect several cells even from a small amount of DP tissue. From these perspectives, frozen–thawed DPSCs can proliferate well and exhibit stemness properties. A substantial number of DP cells can be isolated even from “small” DP tissue, as the tissue continuously provides cells. These features of SCIP are highly appealing, given its high success rate in primary cultures of DP tissue. However, since only 17 samples were used in this study, this method needs to be further refined to optimize the large-scale production of SHEDs while preserving their stem cell properties over the long term.

## 5. Conclusions

In this study, we developed a novel technique (SCIP) for isolating a large number of DP cells through the one-step cultivation of a single DP tissue. This method relies on the outgrowth of cells that migrate from the DP tissue fixed to the plastic surface of a tissue culture dish, followed by their collection via trypsinization, the cryopreservation of isolated cells, and re-cultivation. This cycle of cell cultivation, harvesting, and cryopreservation can be repeated at least ten times, allowing for the collection of DP cells enriched with stem cells. The SCIP method offers several advantages over previous techniques, including the following: (1) no need for special equipment, as only a scalpel is required to rub the samples onto the culture dish, (2) a simple operational protocol with minimal variation among operators, (3) reduced cell damage due to the shorter enzymatic digestion time, (4) the easy cryopreservation of cells harvested by trypsinization, and (5) a high success rate for propagating DP cells from patients with genetic diseases. Additionally, this approach can be potentially adapted for permanent DP and wisdom teeth to obtain a significant number of undifferentiated cells. Thus, the SCIP method is an effective tool for generating a large number of DP cells for both research and clinical applications in the field of regenerative medicine.

## Figures and Tables

**Table 1:** RT-PCR primer sets.

Target Gene	Forward Primer (5′-3′)	Reverse Primer (5′-3′)	Size
GAPDH	ACCACAGTCCATGCCATCAC	TCCACCACCCTGTTGCTGTA	452
OCT3/4	CATGGCGGGACACCTGGCTTC	CTGATCTGCTGCAGTGTGGGTT	783
SOX2	AGGACCAGCTGGGCTACCCG	GCGCCGGGGAGATACATGC	320
NANOG	TTGGAAGCTGCTGGGGGAAG	GATGGGAGGAGGGGAGAGGA	193
NESTIN	AGCCCTGACCACTCCAGTTTAG	CCCTCTATGGCTGTTTCTTTCTCT	128
IGF1	CTCTTCAGTTCGTGTGTGGAGAC	CAGCCTCCTTAGATCACAGCTC	134
DSPP	AAAGTGGTGTCCTGGTGCAT	CCTGGATGCCATTTGCTGTG	246
ABCG	ACCATTGCATCTTGGCTGTC	CGATGCCCTGCTTTACCAAA	181
OCN	CCCTTTCTCCTGTCCGGATG	GCTGAGCTCTAGGGGAGTC	246
CD90	ATGAACCTGGCCATCAGCA	GTGTGCTCAGGCACCCC	218
MSX1	CGCTCGGCCATTTCTCGGTG	CGCTCCAGCGCCAGCAGCTGC	226
MSX2	CTGGTGAAGCCCTTCGAGAC	GGCGTGCGCGGCTTCCGATTG	190

**Table 2:** Summary of success rates of the SCIP method *.

Number	Systemic Disease	Dental Disease	Success/ Failed	Repeated Passages	Stock Tubes
1	Hypophosphatasia	Falling off	Success	6	41
2	Hypophosphatasia	Pulpitis	Success	6	38
3	Osteogenesis Imperfecta	Prolonged Retention	Success	6	43
4	Baller–Gerold Syndrome	Prolonged Retention	Failed	-	-
5	Rett Syndrome	Prolonged Retention	Success	5	31
6	Hypophosphatasia	Falling Off	Success	5	34
7	Hypophosphatasia	Occlusal Trauma	Success	5	32
8	Charge Syndrome	Pulpitis	Success	5	23
9	Charge Syndrome	Pulpitis	Success	5	27
10	Charge Syndrome	Pulpitis	Success	5	28
11	Hypophosphatasia	Prolonged Retention	Success	10	50
12	Health	Falling Off	Success	15	23
13	Osteogenesis Imperfecta	Prolonged Retention	Success	12	26
14	Hypophosphatasia (Adult)	Prolonged Retention	Success	15	43
15	Health	Prolonged Retention	Success	10	27
16	Health	Prolonged Retention	Success	10	27
17	Health	Pulpitis	Success	10	28
		16/17 Samples Succeed (>94% Success Rate)			

* The success or failure of isolating DP cells using SCIP, as well as the history of patients with “systemic diseases” and “dental diseases” (from which DP cells were isolated), are shown. In this table, “Passage” meaning the numbers of repeated passages. “Stock tubes” refers to the number of cryopreserved cell stocks.

**Table 3:** Comparison between the SCIP and conventional method *.

Property	Conventional Method	SCIP Method
Cell fixation	Dependent on DP volume	Possible with small amounts of DP
Continuous supply of primary cells	Not possible (only at once time of cell seeding)	Can be propagated from scratched pulp
Cell proliferation	Varies	Good
Methodological simplicity	Easy	Easy
Difference between operators	Large	Small
Damage to cells	Enzymatic treatment is required for about 1 h	Enzymatic treatment is required for about 20 min
Reuse of DP	Only once time for culture	Can be used repeatedly

* The SCIP method is considered to exhibit several advantages over conventional methods. The considered advantages of the SCIP method are underlined.

## Data Availability

The original contributions presented in the study are included in the article, and further inquiries can be directed to the corresponding author.
